# Identification of a 9-gene autophagy-related signature for predicting prognosis and immune exhaustion features in breast cancer

**DOI:** 10.1007/s13205-026-04756-5

**Published:** 2026-03-10

**Authors:** Jianan Zhang, Yijun Wang, Mengsha Zou

**Affiliations:** Department of Breast and Thyroid Surgery, The Affiliated Lihuili Hospital of Ningbo University, Ningbo, 315040 Zhejiang P. R. China

**Keywords:** Breast cancer, Autophagy, Prognostic signature, Immune exhaustion, Drug sensitivity, Bioinformatics

## Abstract

To address the prognostic limitations in breast cancer (BRCA), we integrated transcriptomic profiles from The Cancer Genome Atlas (TCGA) and Gene Expression Omnibus (GEO) to construct a novel 9-gene autophagy-related signature via Least Absolute Shrinkage and Selection Operator (LASSO) Cox regression. The model demonstrated robust predictive accuracy for overall survival in the training cohort (HR = 2.28, *P <* 0.001) and maintained stability in external validation (AUC = 0.740). Mechanistically, the risk score was significantly associated with selective autophagy pathways and an immune-exhausted microenvironment characterized by T-cell dysfunction. Furthermore, drug sensitivity profiling identified a positive correlation between the risk gene MTDH and sensitivity to Vincristine and Gemcitabine. This study presents a reliable risk-stratification tool that bridges autophagic mechanisms with personalized chemotherapy guidance.

**Supplementary Information:**

The online version contains supplementary material available at 10.1007/s13205-026-04756-5.

## Introduction

Breast cancer (BRCA) has surpassed lung cancer as the most frequently diagnosed malignancy worldwide, posing a tremendous challenge to global public health(Sung et al. 2021; Fumagalli and Barberis 2021). According to recent global cancer statistics, BRCA remains the leading cause of cancer-related mortality among women, with incidence rates continuing to rise in both developed and developing regions(Baliu-Piqué et al. 2020). Currently, the standard of care involves a multimodal approach comprising surgical resection, chemotherapy, radiotherapy, endocrine therapy, and targeted biological agents (e.g., anti-HER2 therapies) (Shenkier 2004; Grunfeld 2005; Greenlee et al. 2017). Despite significant advancements in these therapeutic strategies, the clinical outcomes of BRCA patients remain highly heterogeneous. Traditional clinicopathological indicators, such as TNM staging, histological grade, and molecular subtyping (ER, PR, HER2 status), are widely used for risk stratification but often fail to fully capture the intricate biological complexity of the disease (Benson 2003; Cserni et al. 2018). Consequently, a substantial proportion of patients with similar clinical features experience vastly different prognoses, with many suffering from unexpected recurrence, distant metastasis, and the development of multi-drug resistance (Tran et al. 2018; Afzal and Vahdat 2024). This persistent clinical dilemma underscores an urgent need to identify novel, robust molecular biomarkers that can transcend traditional staging systems to optimize risk assessment and guide personalized precision medicine (Meehan et al. 2020; Afzal and Vahdat 2024).

In the context of tumorigenesis, autophagy functions as a complex “double-edged sword,” a duality well-documented in breast cancer research(Gu et al. 2016; Chavez-Dominguez et al. 2020; Klionsky et al. 2021). On one hand, autophagy acts as a tumor suppressor in early neoplastic transformation. For instance, the monoallelic deletion of the essential autophagy gene BECN1 (Beclin 1) is observed in 40–75% of breast cancers, where its loss promotes genomic instability and accelerates mammary tumorigenesis (Liang et al. 1999, p. 1; Yu et al. 2024b). However, in established tumors, the role of autophagy often shifts towards promoting survival and therapeutic resistance. Cancer cells hijack this mechanism to mitigate metabolic stress and evade cytotoxicity. Specific examples include the observation that protective autophagy mediates acquired resistance to Tamoxifen in ER-positive breast cancer by preventing apoptosis (Samaddar et al. 2008), and facilitates survival in HER2-positive tumors treated with Trastuzumab (Triulzi et al. 2018). Similarly, in triple-negative breast cancer (TNBC), elevated autophagic flux has been linked to reduced sensitivity to taxane-based chemotherapies like Paclitaxel, where autophagy inhibitors have been shown to restore drug efficacy (Luo et al. 2016). Given this pivotal role, ARGs hold immense promise as potential biomarkers, yet their systemic integration into prognostic models remains to be fully explored(Jin et al. 2022; Yu et al. 2024a).

Despite the revolutionary success of immune checkpoint inhibitors (ICIs) in melanoma and lung cancer, their efficacy in breast cancer remains limited. Most breast tumors exhibit an immunologically ‘cold’ phenotype characterized by low T-cell infiltration and intrinsic resistance to PD-1/PD-L1 blockade (Emens 2018). Therefore, deciphering the molecular mechanisms that drive immune exclusion and exhaustion is a top priority. Recent research has highlighted a dynamic crosstalk between autophagy, the tumor microenvironment (TME), and drug sensitivity. Dysregulated autophagy can reshape the immune landscape, often leading to an immunosuppressive microenvironment. A seminal study demonstrated that pancreatic cancer cells utilize selective autophagy mediated by the receptor NBR1 to degrade MHC-I molecules, thereby impairing antigen presentation and allowing tumors to evade CD8 + T cell killing (Yamamoto et al. 2020). In the context of breast cancer, autophagy has also been implicated in regulating the stability of the immune checkpoint protein PD-L1, directly influencing the efficacy of immunotherapy (Clark et al. 2017). Furthermore, metabolic stress in the TME can induce maladaptive autophagy in infiltrating T cells, contributing to a state of “exhaustion” where T cells lose their effector functions and upregulate inhibitory receptors (Wherry 2011). Despite these isolated findings, few studies have successfully constructed a unified model that links ARG expression patterns simultaneously to immune exhaustion status and chemotherapeutic sensitivity (Xia et al. 2021; Jin et al. 2022). Therefore, identifying a robust signature that can bridge these biological mechanisms with clinical precision medicine is essential for advancing breast cancer management(Deng et al. 2021). Although autophagy is known to modulate immune responses, systematic studies linking autophagy-related gene signatures directly to the ‘immune-exhausted’ phenotype in breast cancer are lacking. Current prognostic models rarely integrate these two critical biological processes to guide immunotherapy.

In the present study, we systematically analyzed the expression profiles of ARGs in BRCA using large-scale transcriptomic data from The Cancer Genome Atlas (TCGA) and Gene Expression Omnibus (GEO) databases. We constructed a robust 9-gene autophagy-related prognostic signature via LASSO Cox regression analysis. Beyond prognostic prediction, we extensively investigated the association between the signature and the immune landscape, revealing a potential mechanism of immune exhaustion in high-risk patients. Furthermore, we screened for potential sensitive drugs targeting high-risk patients, providing new insights for precision medicine and therapeutic decision-making in breast cancer.

## Materials and methods

### Data collection and preprocessing

Publicly available RNA-sequencing (RNA-seq) data (HTSeq-FPKM) and corresponding clinical information of Breast Invasive Carcinoma (BRCA) were obtained from The Cancer Genome Atlas (TCGA) database (https://portal.gdc.cancer.gov/) (The Cancer Genome Atlas Research Network et al. 2013), serving as the training cohort. For external validation, an independent microarray dataset [GSE20685] was retrieved from the Gene Expression Omnibus (GEO) database (https://www.ncbi.nlm.nih.gov/geo/) (Barrett et al. 2012). The raw data were normalized and log2-transformed to ensure data comparability. Additionally, a comprehensive list of 232 ARGs was downloaded from the Human Autophagy Database (HADb, http://www.autophagy.lu/) (Moussay et al. 2011).

### Identification of differentially expressed autophagy-related genes (DE-ARGs)

Differential expression analysis between breast cancer tissues and normal tissues was performed using the limma package inR software (version 4.4.2) (Ritchie et al. 2015). The selection criteria were set as a False Discovery Rate (FDR) < 0.05 and |log2 Fold Change| > 1. The identified DEGs were then intersected with the ARG list from HADb to obtain the DE-ARGs.

### Screening of prognostic candidates

To screen for genes with significant prognostic value, the Kaplan-Meier Plotter database (https://kmplot.com/analysis/) was utilized (Győrffy 2021). The identified DE-ARGs were entered into the system, and the “auto select best cutoff” option was applied to stratify patients. Genes with a Log-rank *P* < 0.05 in the Overall Survival (OS) analysis were considered potential prognostic candidates and selected for further modeling.

### Construction and validation of the prognostic signature

To construct a robust prognostic model and minimize the risk of overfitting, Least Absolute Shrinkage and Selection Operator (LASSO) Cox regression analysis was performed using the glmnet package in R(Friedman et al. 2010). We employed a 10-fold cross-validation strategy to optimize the penalty parameter (λ). Specifically, the training dataset was randomly partitioned into 10 non-overlapping subsets. In each iteration, the model was trained on nine subsets and validated on the remaining one to calculate the partial likelihood deviance. This process was repeated 10 times to ensure the stability of the results(Lin and Zelterman 2002). The optimal λ value was ultimately determined based on the minimum partial likelihood deviance criteria λ min, yielding the most parsimonious model with the best predictive performance.

The risk score for each patient was calculated using the following formula:

$${\text{Risk~Score = }}\mathop \sum \limits_{{{\text{i = 1}}}}^{{\mathrm{n}}} \left( {{\mathrm{Coef}}_{{\mathrm{i}}} \times {\mathrm{Exp}}_{{\mathrm{i}}} } \right)$$

Where Coef$\:\mathrm{i}$,represents the regression coefficient derived from the multivariate Cox analysis, and Exp$\:\mathrm{i}$ represents the normalized expression value of gene $\:\mathrm{i}$. Patients were categorized into high- and low-risk groups based on the median risk score. Kaplan-Meier (K-M) survival curves and Time-dependent Receiver Operating Characteristic (ROC) curves were generated using the survival and timeROC packages (Blanche et al. 2013) to evaluate the predictive performance in both the TCGA training cohort and the GEO validation cohort.

### Functional enrichment and ppi network analysis

To explore the biological functions of the signature genes, Gene Ontology (GO) and Kyoto Encyclopedia of Genes and Genomes (KEGG) pathway enrichment analyses were performed using Metascape (https://metascape.org/) (Zhou et al. 2019), with a significance threshold of *P* < 0.01. A Protein-Protein Interaction (PPI) network was constructed using the STRING database (version 11.5, https://string-db.org/) (Szklarczyk et al. 2019) with a minimum required interaction score of 0.4.

### Assessment of independent prognostic factors

To evaluate whether the 9-gene signature serves as an independent prognostic factor for breast cancer, univariate and multivariate Cox proportional hazards regression analyses were performed in the TCGA cohort. The risk score was analyzed alongside available clinicopathological characteristics, including patient age, pathologic T stage, pathologic N stage, and ERBB2 status. The hazard ratio (HR) and 95% confidence interval (CI) were calculated to quantify the risk association. Factors with a *P <* 0.05 in the multivariate analysis were considered statistically significant independent prognostic indicators.

### Immune infiltration and drug sensitivity analysis

The immune landscape was analyzed using the GSCALite platform (http://bioinfo.life.hust.edu.cn/web/GSCALite/) (Liu et al. 2018). The correlation between the signature-based Gene Set Variation Analysis (GSVA) score and the infiltration levels of diverse immune cells was evaluated. Furthermore, the drug sensitivity of the signature genes was assessed based on data from the Genomics of Drug Sensitivity in Cancer (GDSC) (Yang et al. 2012) and Cancer Therapeutics Response Portal (CTRP) (Rees et al. 2016) databases via GSCALite. Spearman correlation analysis was performed to identify the association between gene expression and the half-maximal inhibitory concentration (IC50) of therapeutic agents.

### Protein level validation

To verify the expression of the identified prognostic genes at the translational level, immunohistochemistry (IHC) staining images were retrieved from the Human Protein Atlas (HPA) database (https://www.proteinatlas.org/). We selected representative key risk genes, including MTDH, HSP90AA1, and VDAC1, to compare their protein expression patterns between normal breast tissues and breast invasive carcinoma tissues. The staining intensity was visually evaluated to validate the transcriptomic analysis results.

### Statistical analysis

All statistical analyses were performed usingR software (version 4.4.2). Differences in gene expression were analyzed using the Wilcoxon test. Survival differences were compared using the Log-rank test. *P* < 0.05 was considered statistically significant.

## Result

### Clinical characteristics of the study cohorts

The comprehensive clinical annotations of the training set (TCGA-BRCA, *n* = 1083) and the external validation set (GSE20685, *n* = 102) were analyzed. In the TCGA cohort, the median age was 58 years, with 53.8% of patients aged 60 years. The majority of patients were diagnosed with early-stage breast cancer (Stage I-II, approx. 72%), while Stage III and IV accounted for 23.1% and 1.8%, respectively. Similarly, the external validation cohort (GSE20685) consisted of 102 breast cancer patients with comparable baseline characteristics. These clinical features were incorporated into the multivariate regression analysis to assess the independence of the prognostic signature.

### Identification of differentially expressed autophagy-related genes in breast cancer

**Fig. 1:**
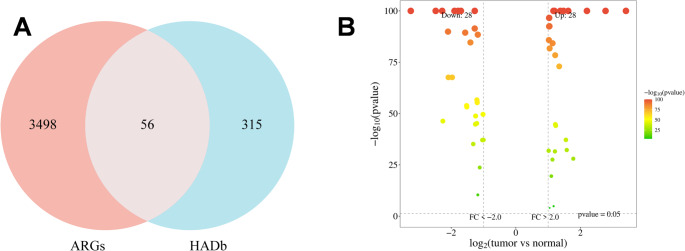
Identification of DE-ARGs in breast cancer.**A** Venn diagram illustrating the intersection between DEGs identified in the TCGA-BRCA cohort and ARGs retrieved from the HADb database. **B** Volcano plot visualizing the differential expression patterns of the 56 candidate DE-ARGs between tumor and normal tissues

To identify candidate biomarkers involved in breast cancer progression, we obtained transcriptome RNA-sequencing data from the TCGA database. A total of 232 ARGs were retrieved from the HADb database. We performed differential expression analysis between tumor and normal tissues using the limma algorithm. To ensure the identified genes possessed both statistical significance and biological magnitude, stringent filtering criteria were applied (|log2FC| > 1 and FDR < 0.05). As a result, 56 genes were identified as DE-ARGs, visualized in the Venn diagram (Fig. 1A). The expression distribution of these genes was further illustrated via a volcano plot (Fig. 1B), showing 28 upregulated and 28 downregulated genes in tumor tissues. These differentially expressed genes served as the initial pool for prognostic modeling.

### Screening of prognostic autophagy-related genes via Kaplan-Meier survival analysis

**Fig. 2:**
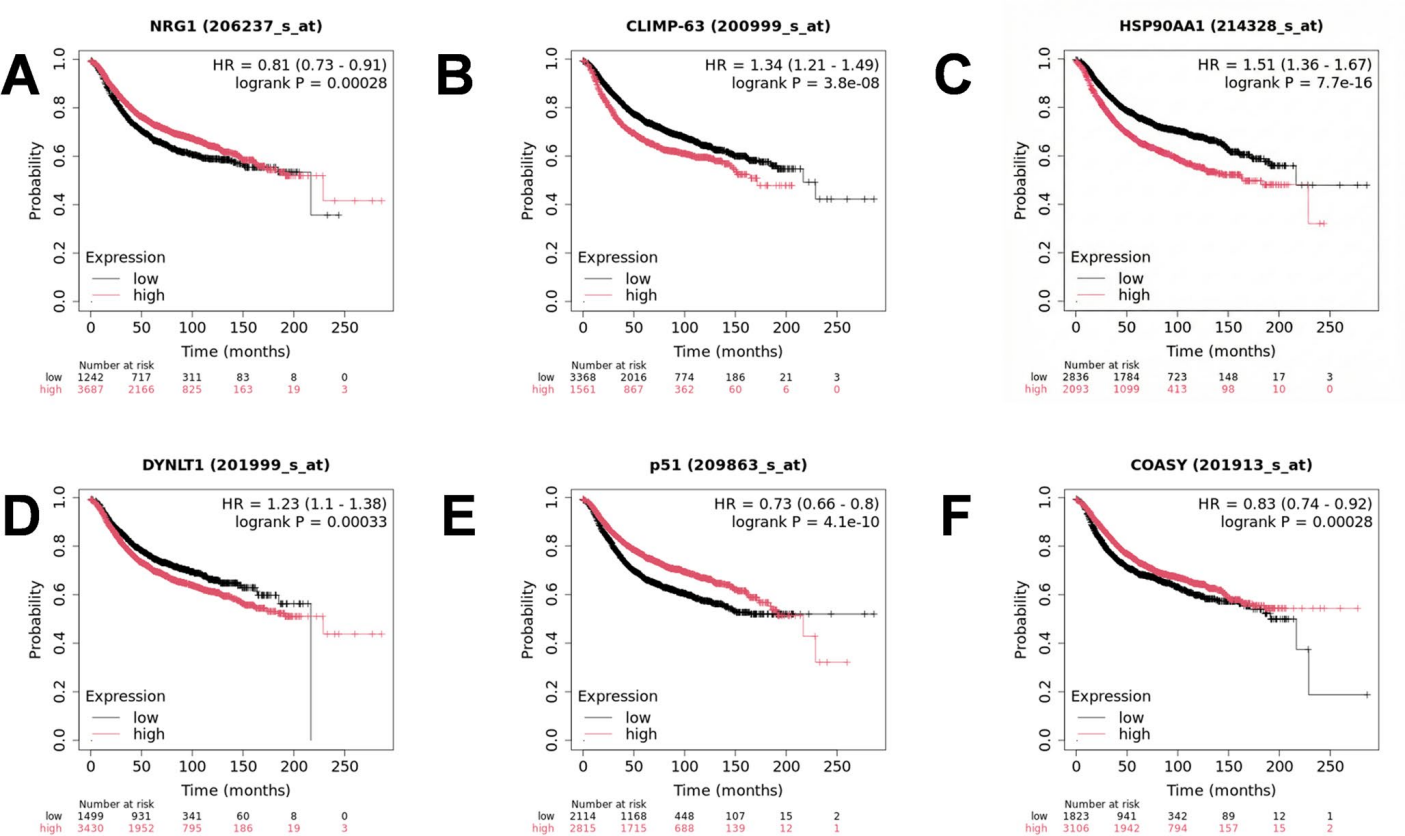
Kaplan-Meier survival analysis of representative prognostic DE-ARGs in breast cancer. **A**–**C** Kaplan-Meier curves of three representative genes identified as risk factors. **D**–**F** Kaplan-Meier curves of three representative genes identified as protective factors. The overall survival (OS) analysis was performed using the Kaplan-Meier Plotter database based on mRNA gene expression. Patients were stratified into high- and low-expression groups based on the auto-selected best cutoff values. The red line indicates the high-expression group, and the black line indicates the low-expression group

To further narrow down the candidates to those with genuine prognostic value, we performed survival analysis using the Kaplan-Meier Plotter database. The Log-rank test was utilized to statistically evaluate the significant difference in Overall Survival (OS) between high- and low-expression groups. As shown in Fig. 2, we successfully identified candidate genes significantly associated with patient outcomes. For instance, high expression of HSP90AA1 (HR = 1.51, *P* < 0.001) was identified as a significant risk factor, whereas TP63 (HR = 0.73, *P* < 0.001) functioned as a protective factor. Genes with a significant Log-rank p 0.05 were retained for the subsequent construction of the multivariate model.To comprehensively screen for potential prognostic biomarkers and maximize the sensitivity of identification, the ‘auto select best cutoff’ algorithm was employed for single-gene survival analysis in the Kaplan-Meier Plotter database. This strategy calculates all possible percentiles between the lower and upper quartiles and selects the threshold with the highest statistical significance, thereby avoiding the omission of potential candidates due to rigid cutoff values.

### Construction and evaluation of a 9-gene autophagy-related prognostic signature

**Fig. 3:**
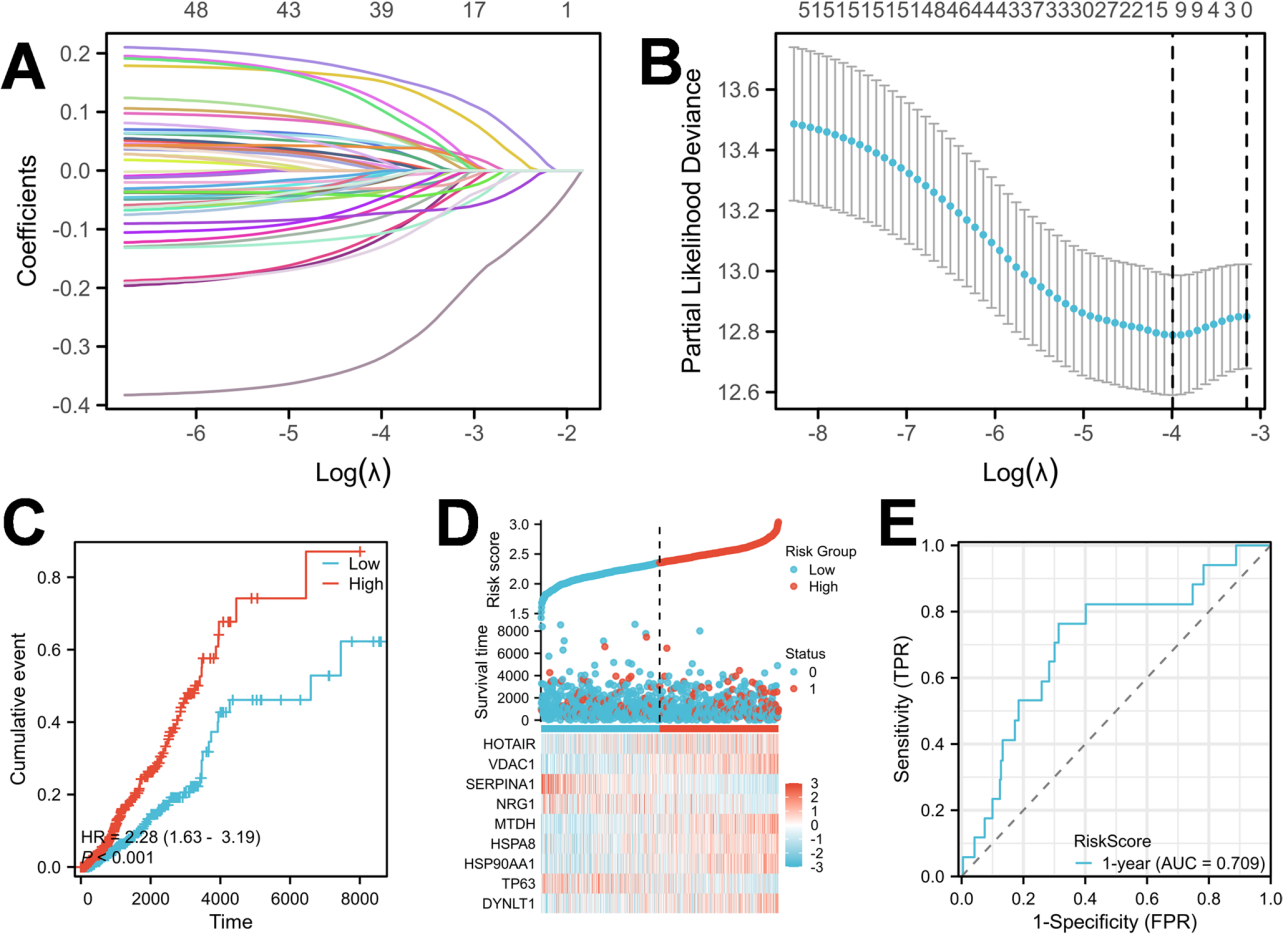
Construction and evaluation of the 9-gene autophagy-related prognostic signature in the TCGA training cohort. **A** LASSO coefficient profiles of the 56 candidate autophagy-related genes. Each curve represents the coefficient of a single gene changing with the penalty parameter. **B** Selection of the optimal penalty parameter in the LASSO Cox regression model using 10-fold cross-validation. The vertical dashed lines indicate the optimal used to define the 9-gene signature. **C** Kaplan-Meier survival analysis of Overall Survival (OS) between high- and low-risk groups. **D** Risk factor analysis illustrating the distribution of risk scores (top), the survival status of patients (middle, where red dots indicate death), and the expression heatmap of the 9 signature genes (bottom). The vertical dotted line represents the median risk score cutoff distinguishing high- and low-risk groups. **E** Time-dependent ROC curve for predicting 1-year survival in the training cohort

To resolve multicollinearity among the prognostic candidates and prevent model overfitting, we employed the Least Absolute Shrinkage and Selection Operator (LASSO) Cox regression algorithm. The trajectory of regression coefficients is shown in Fig. 3A. The optimal penalty parameter (λ) was selected via 10-fold cross-validation (Fig. 3B, Table S1 and Table S2), ultimately identifying a robust 9-gene signature (HOTAIR, VDAC1, SERPINA1, NRG1, MTDH, HSPA8, HSP90AA1, TP63, and DYNLT1).A risk score was then calculated for each patient based on the regression coefficients. To evaluate the clinical relevance of the model, patients were categorized into high- and low-risk groups. Kaplan-Meier analysis demonstrated that the high-risk group had significantly poorer OS (HR = 2.28, *P* < 0.001), confirming the discriminative ability of the risk score (Fig. 3C). Furthermore, to assess the predictive accuracy (sensitivity and specificity) of the signature, we performed Time-dependent ROC analysis using the timeROC method. The Area Under the Curve (AUC) for 1-year survival was 0.709 (Fig. 3E), indicating satisfactory predictive performance in the training cohort.To ensure the robustness and reproducibility of the prognostic model and prevent overfitting, patients were categorized into high- and low-risk groups based on the median risk score. Unlike the optimal cutoff used in the screening phase, the median serves as an unbiased and standardized threshold for the multivariate signature, ensuring balanced sample sizes in both groups for rigorous statistical comparison.

### Validation of the prognostic signature in an external independent cohort

**Fig. 4:**
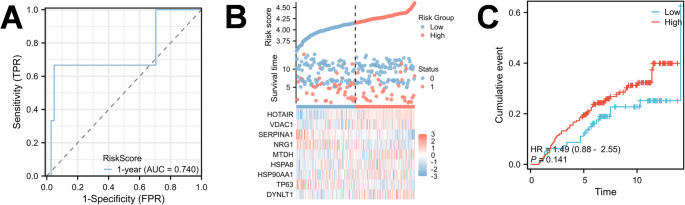
Validation of the 9-gene prognostic signature in the independent GEO external cohort. **A** Time-dependent ROC curve analysis for predicting 1-year survival in the GEO validation cohort. **B** Risk factor analysis in the validation cohort, showing the distribution of risk scores (top), survival status (middle), and the expression heatmap of the signature genes (bottom). **C** Kaplan-Meier survival analysis in the validation cohort

To verify the robustness and generalization ability of the model, we applied the same formula to an independent external dataset (GSE20685) from the GEO database. The predictive accuracy was first evaluated using ROC analysis. Remarkably, the signature maintained excellent performance with an AUC of 0.740 (Fig. 4A), suggesting that the model is robust across different sequencing platforms and populations. Consistent with the training set, the distribution of risk scores and gene expression patterns (Fig. 4B) confirmed that risk-associated genes were upregulated in high-risk patients. Although the survival difference in the validation cohort did not reach statistical significance (*P* = 0.141) likely due to limited sample size, a distinct trend of poorer survival in the high-risk group was observed (HR = 1.49), further supporting the validity of our signature (Fig. 4C).

### Functional enrichment analysis of the signature

**Fig. 5:**
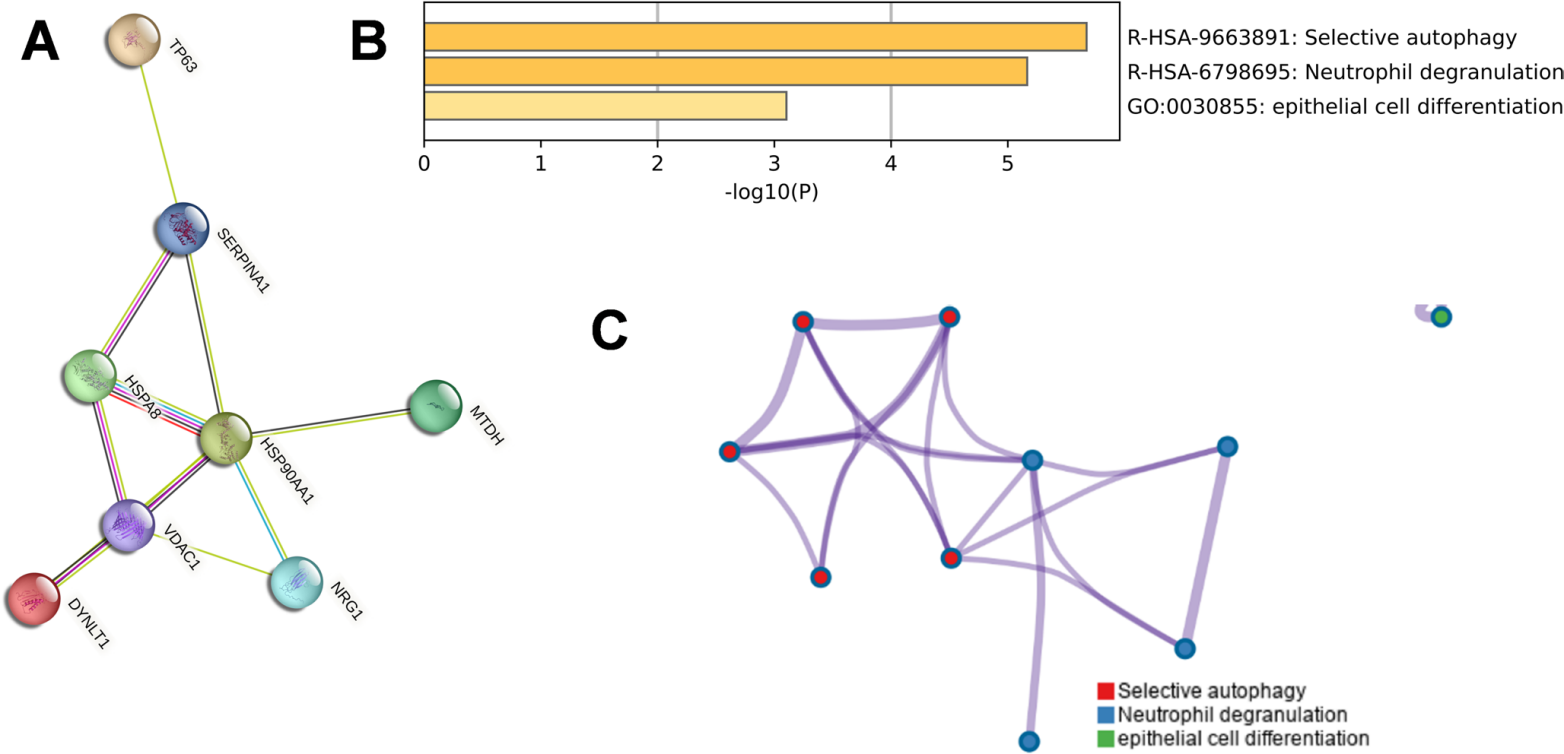
Protein-Protein Interaction (PPI) network and functional enrichment analysis of the prognostic signature. **A** PPI network of the 8 protein-coding signature genes constructed using the STRING database. **B** Bar graph of the top enriched biological processes and pathways identified by Metascape analysis. **C** Network visualization of the enriched ontology terms, illustrating the inter-cluster relationships and the close functional connection between autophagy and immune-related processes. Isolated nodes in the PPI network indicate proteins that do not show direct interactions with the main cluster (confidence score > 0.4), suggesting they may function through independent pathways

To evaluate the physical connectivity and potential functional crosstalk among the prognostic genes, a Protein-Protein Interaction (PPI) network was constructed using the STRING database. Given that HOTAIR is a long non-coding RNA (lncRNA) and does not encode a protein, the network analysis focused on the eight protein-coding genes. As illustrated in Fig. 5A, the topological structure of the network revealed a dense interaction cluster anchored by the molecular chaperones HSP90AA1 and HSPA8. These central hub nodes exhibited strong connectivity with VDAC1, SERPINA1, and NRG1, indicative of a coordinated functional module governing protein folding and stress responses. Conversely, TP63 and MTDH appeared as isolated nodes, suggesting that these genes may exert their prognostic effects through independent mechanisms distinct from the chaperone complex.To further validate the biological relevance of the signature and decipher the underlying signaling pathways, we performed functional enrichment analysis using Metascape. This step aimed to verify whether the identified genes effectively represent autophagy-specific alterations. The results confirmed that the prognostic signature is intrinsically linked to autophagy regulation. As shown in Fig. 5B, “Selective autophagy” (R-HSA-9663891) emerged as the most significantly enriched ontology term, followed by “Neutrophil degranulation” and “Epithelial cell differentiation.” The dominance of selective autophagy serves as a robust validation of our model’s specificity. Furthermore, the network visualization of enriched terms (Fig. 5C) highlighted the inter-cluster relationships, elucidating a potential regulatory axis connecting autophagic processes with immune responses.

### Immune landscape characterization and drug sensitivity analysis

**Fig. 6:**
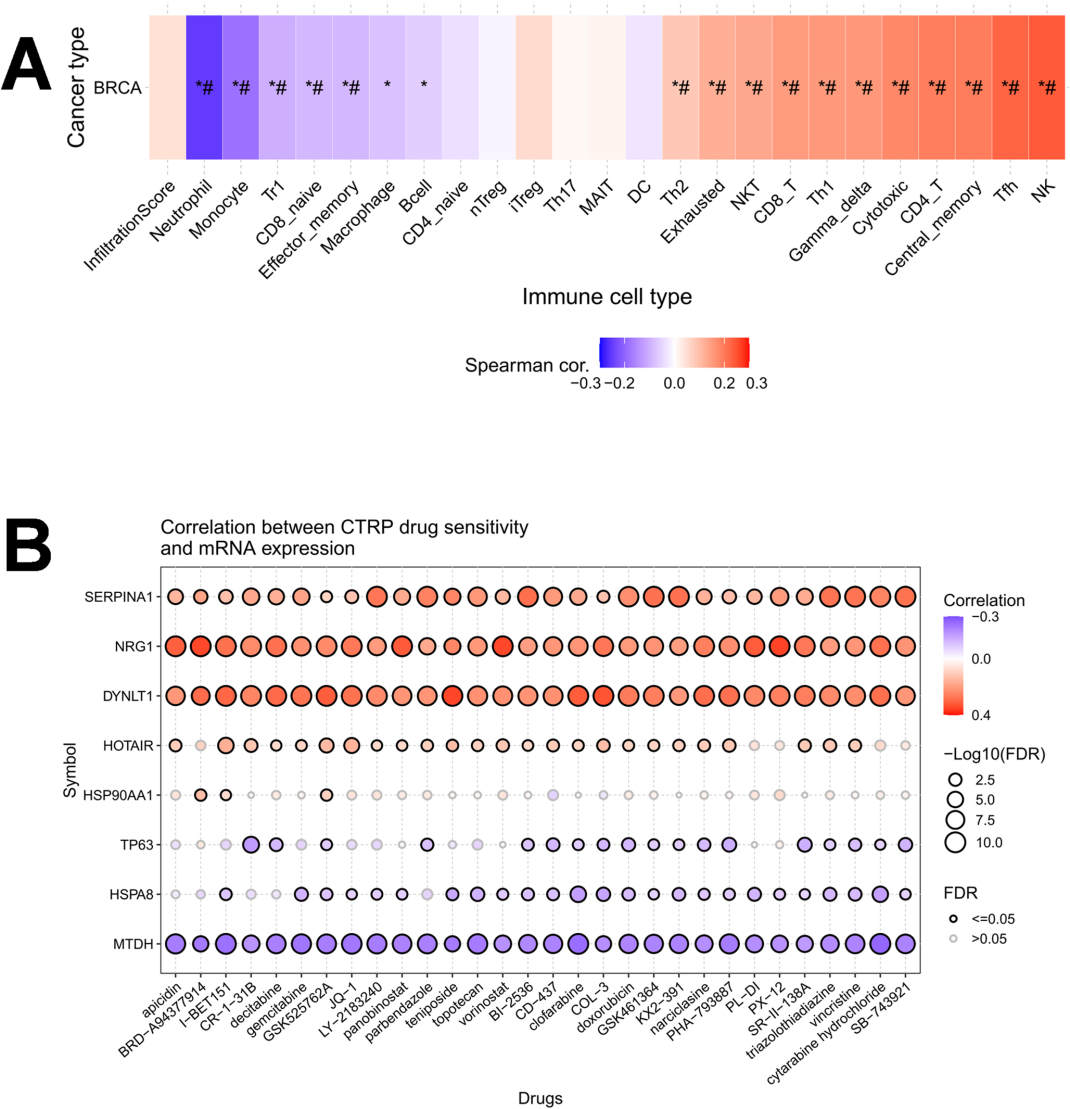
Immune landscape characterization and drug sensitivity analysis. **A** Heatmap illustrating the Spearman correlation between the prognostic signature (Risk Score) and immune cell infiltration levels in breast cancer (GSCALite). Red blocks indicate positive correlations, while blue blocks indicate negative correlations. **B** Bubble heatmap showing the correlation between the expression of the 9 signature genes and drug sensitivity (IC50 values) from the CTRP database. Blue bubbles represent negative correlations (higher gene expression is associated with lower IC50, indicating higher drug sensitivity), while red bubbles represent positive correlations (drug resistance)

To dissect the heterogeneity of the tumor microenvironment (TME) driven by the prognostic signature, we evaluated the association between the signature-derived GSVA scores and immune cell infiltration patterns using the GSCALite platform. This analysis aimed to determine whether the prognostic risk is modulated by specific immune evasion mechanisms. As visualized in the correlation heatmap (Fig. 6A), the signature exhibited significant associations with distinct immune cell subsets in breast cancer. Notably, the risk score displayed a strong positive correlation with Th2 cells, Cytotoxic cells, and Exhausted T cells (indicated by red clusters), while showing a negative correlation with Neutrophils and Monocytes (blue clusters). The paradoxical enrichment of both cytotoxic and exhausted T cells suggests a state of immune exhaustion: although effector lymphocytes are recruited to the tumor site in high-risk patients, they likely enter a dysfunctional state, thereby failing to exert effective anti-tumor immunity. This immunosuppressive feature provides a plausible biological explanation for the poorer prognosis observed in the high-risk group.

Furthermore, to translate these molecular findings into clinical therapeutic strategies, we assessed the correlation between signature gene expression and drug sensitivity (IC50 values) using the CTRP database. The objective of this analysis was to identify potential resistance mechanisms and therapeutic vulnerabilities. The bubble heatmap (Fig. 6B) revealed a polarized landscape of drug response. Genes such as SERPINA1 and NRG1 showed broad positive correlations with IC50 values (red bubbles), implying that their upregulation may confer multidrug resistance. Conversely, MTDH exhibited significant negative correlations with the IC50 of several agents (blue bubbles), including Vincristine, Vorinostat, and Gemcitabine. These findings suggest a stratified therapeutic approach: while high-risk tumors driven by MTDH expression may be refractory to standard treatments, they could potentially be targeted effectively with regimens containing these specific agents.

### Evaluation of the independent prognostic value of the signature

**Fig. 7:**
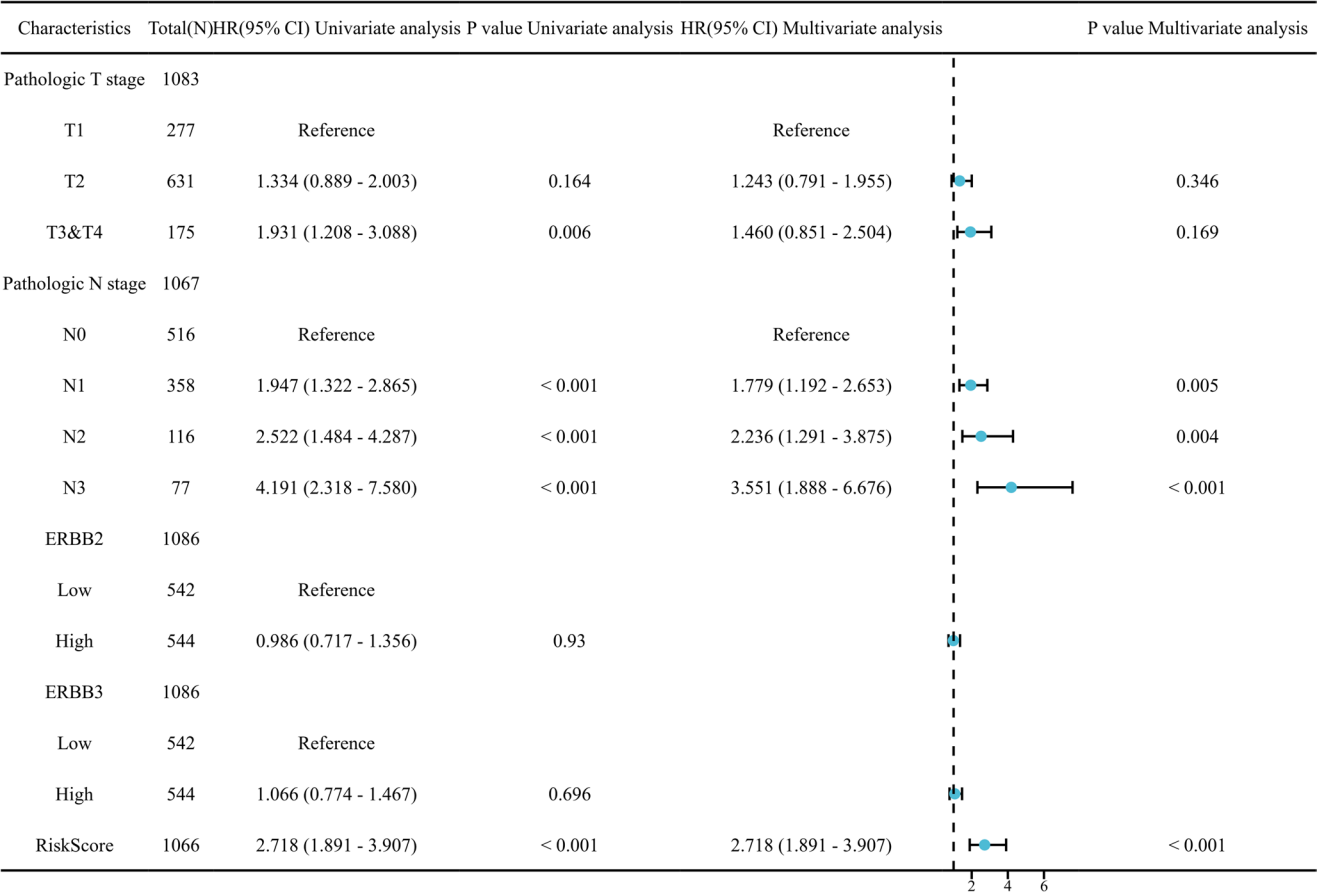
Independent prognostic analysis. Forest plot visualizing the multivariate Cox regression analysis results. The risk score (*P* < 0.001) and N stage serve as independent prognostic factors for overall survival in breast cancer patients

To determine whether the 9-gene signature could serve as an independent prognostic factor for breast cancer patients, we performed univariate and multivariate Cox regression analyses in the TCGA cohort. The analysis included the risk score and accessible clinical characteristics, such as Pathologic T stage, Pathologic N stage, and ERBB2 status. As shown in Table S3 and Fig. 7, the univariate analysis indicated that the risk score was significantly associated with overall survival (HR = 2.718, 95% CI = 1.891–3.907, *P* < 0.001). Importantly, after adjusting for confounding factors in the multivariate analysis, the risk score remained a robust and independent predictor of prognosis (HR = 2.718, 95% CI = 1.891–3.907, *P* < 0.001). These findings suggest that the prognostic value of our signature is independent of traditional clinicopathological features.

### Construction of a prognostic nomogram based on the signature

**Fig. 8:**
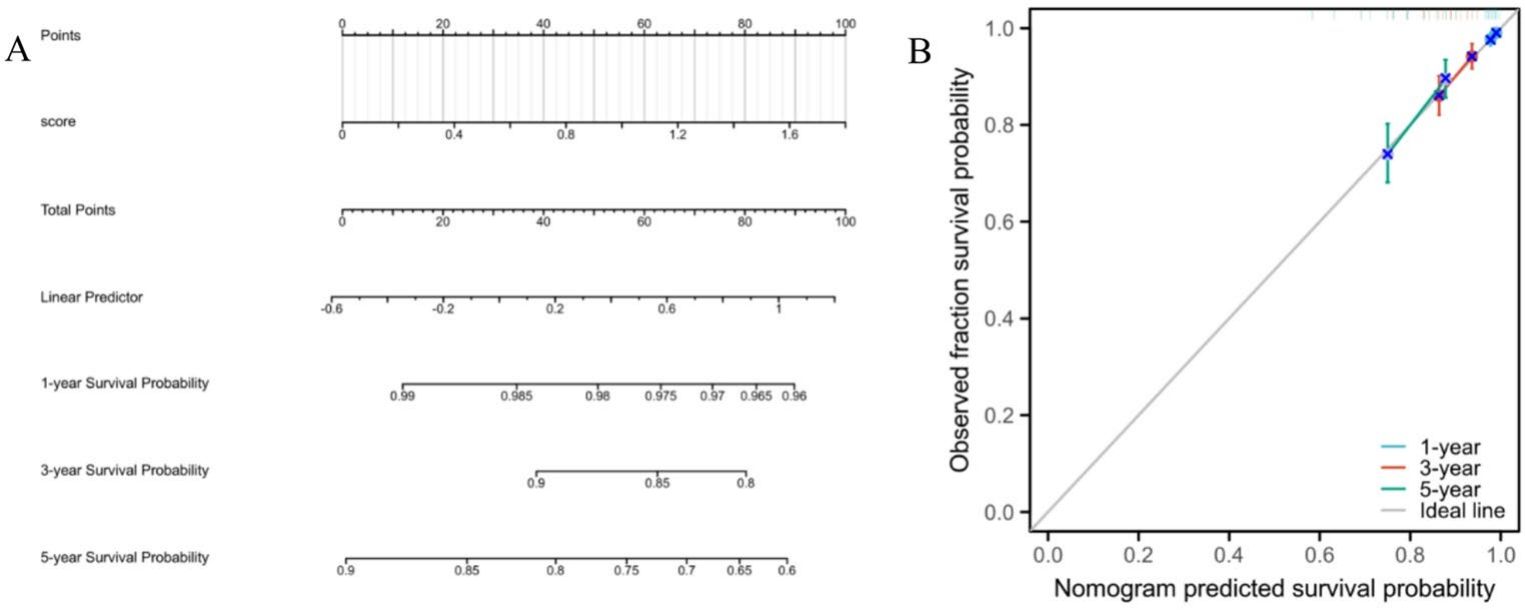
Construction of a prognostic nomogram. A nomogram established based on the 9-gene risk score to predict the 1-, 3-, and 5-year overall survival probability of breast cancer patients. The “Points” scale allows for the conversion of the risk score into a quantitative prognostic metric. **B** Calibration curves of the nomogram for predicting 1-, 3-, and 5-year survival (bootstrap method with 1000 resamples). The x-axis represents the predicted survival probability, and the y-axis represents the actual observed survival probability. The diagonal line represents the ideal prediction

To facilitate the clinical application of our 9-gene signature, we constructed a prognostic nomogram to quantify the predicted survival probability for individual patients (Fig. 8A). In this model, the risk score is mapped to a specific point value on the “Points” scale. By locating the patient’s risk score and identifying the corresponding position on the “Total Points” axis, the estimated 1-, 3-, and 5-year overall survival (OS) probabilities can be determined. As shown in the nomogram, a higher risk score corresponds to a lower survival probability. Furthermore, the predictive accuracy of the nomogram was evaluated using calibration curves (Fig. 8B). The calibration plots for 1-, 3-, and 5-year OS demonstrated excellent agreement between the nomogram-predicted probabilities and the actual observed survival rates, with the curves closely tracking the ideal 45-degree diagonal line. This indicates that the nomogram based on our 9-gene signature possesses high reliability and calibration accuracy.

### Validation of protein expression patterns using the HPA database

**Fig. 9:**
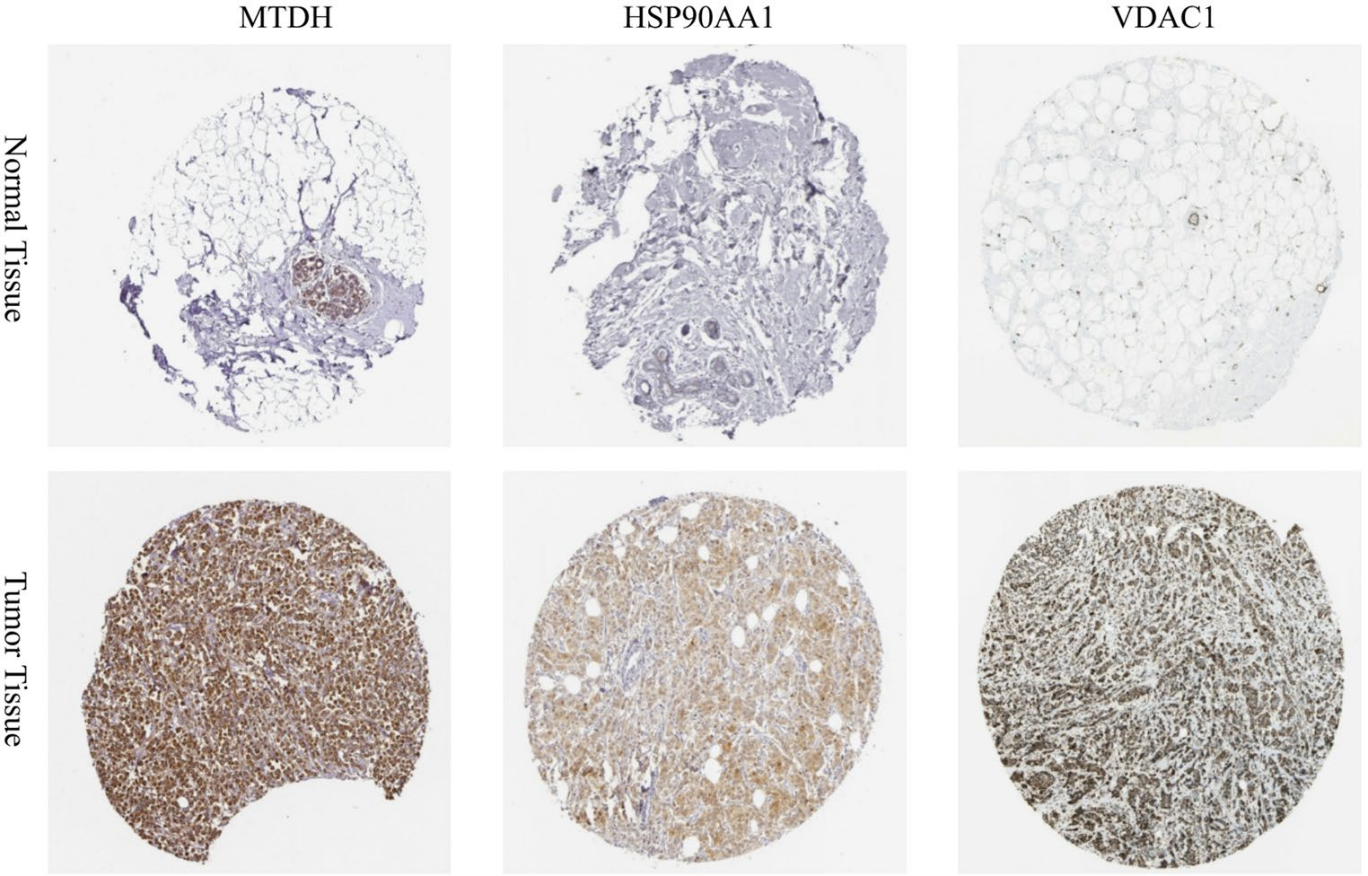
Protein level validation of key signature genes. Representative immunohistochemistry (IHC) staining images of MTDH, HSP90AA1, and VDAC1 in normal breast tissues (upper panel) and breast cancer tissues (lower panel). The images were retrieved from the Human Protein Atlas (HPA) database. The results demonstrate that the protein expression of these risk genes is significantly higher in tumor tissues (strong brown staining) compared to normal tissues (weak or negative staining)

To further corroborate the biological significance of our signature, we validated the protein expression levels of key risk genes using IHC data from the HPA database. As illustrated in Fig. 9, the protein expression levels of MTDH, HSP90AA1, and VDAC1 were markedly upregulated in breast cancer tissues compared to normal tissues. Specifically, normal breast tissues exhibited weak or non-detected staining (mostly visible in adipocytes or sparse ductal cells), whereas tumor tissues displayed moderate to strong brown staining intensity, indicating high protein abundance. These findings are consistent with our mRNA differential expression analysis, confirming that these autophagy-related genes are overexpressed at the protein level in breast cancer.

## Discussion

In this study, we aimed to address the challenge of biological heterogeneity in breast cancer (BRCA) by constructing a robust prognostic tool. By systematically integrating transcriptomic profiles from TCGA and GEO databases, we successfully established a novel 9-gene autophagy-related signature via LASSO Cox regression analysis. Our model demonstrated superior prognostic capacity, effectively stratifying patients into distinct risk groups. Specifically, the high-risk group exhibited a significantly poorer Overall Survival (OS) in the training cohort, with a Hazard Ratio of 2.28 (*P* < 0.001). Importantly, the robustness of this signature was confirmed in an independent external validation cohort, where it achieved a high Area Under the Curve (AUC) of 0.740 for 1-year survival, indicating excellent generalization ability compared to traditional clinicopathological indicators. These quantitative results confirm that our autophagy-based signature serves as a reliable biomarker for risk stratification in BRCA patients.

Our study provides new insights into the molecular mechanisms of BRCA by identifying specific ARGs linked to prognosis. Consistent with previous findings, we identified HOTAIR and MTDH as key risk factors. HOTAIR, a well-known lncRNA, has been reported to promote epithelial-mesenchymal transition (EMT) and metastasis by remodeling chromatin state (Gupta et al. 2010; Mozdarani et al. 2020). Similarly, MTDH is known to facilitate multi-drug resistance and angiogenesis in aggressive breast cancers (Hu et al. 2009). However, unlike previous studies that focused on single biomarkers (Zheng et al. 2018), our study innovatively constructs a multi-gene panel that captures the synergistic effects of these genes. Functional enrichment analysis revealed that our risk score is intimately associated with “selective autophagy” pathways (Gatica et al. 2018). This suggests that high-risk tumors may utilize specific autophagic machinery to recycle nutrients and survive metabolic stress, a mechanism that has been increasingly recognized as a hallmark of advanced malignancy (Jin et al. 2022). It is well-established that autophagy plays a dual role in cancer, functioning as either a tumor suppressor (cytotoxic) or a survival mechanism (cytoprotective) depending on the context. Our results indicate that the high-risk score is strongly associated with poor survival and chemoresistance, suggesting that our signature primarily captures ‘cytoprotective autophagy.’ In this scenario, upregulated autophagy genes help tumor cells mitigate metabolic stress and recycle nutrients within the hypoxic tumor microenvironment, thereby shielding them from apoptotic cell death. This maladaptive survival mechanism explains why high-risk patients exhibit aggressive clinical features and refractory responses to standard therapies. By systematically linking these genes to selective autophagy, our model offers a more comprehensive biological interpretation than single-gene studies.

A major novelty of this study lies in bridging the critical gap between autophagy-based prognosis, immune microenvironment characterization, and precision chemotherapy. We observed a distinct “immune-exhausted” landscape in high-risk patients, characterized by the enrichment of exhausted T cells and depletion of neutrophils(DeNardo and Coussens 2007; Wherry 2011). While previous literature suggests that dysregulated autophagy can impair antigen presentation (Yamamoto et al. 2020; Wang et al. 2025), we further propose that a “metabolic competition” mechanism may drive this immunosuppression. Genes such as HSP90AA1 stabilize proteins to support high metabolic rates in tumor cells under stress. The hyper-activation of autophagy likely enhances tumor metabolic fitness, leading to the rapid depletion of glucose and essential nutrients in the tumor microenvironment (TME). Since effector T cells are highly dependent on glycolysis for function, this nutrient-deprived environment forces them into metabolic insufficiency, thereby driving them toward an exhausted phenotype (Yi et al. 2010). At the molecular level, our signature captures complex regulatory networks, exemplified by the inclusion of HOTAIR, a long non-coding RNA. Its inclusion in the Human Autophagy Database is justified by its role as a critical upstream regulator rather than a direct executioner. Accumulating evidence indicates that lncRNAs function as competing endogenous RNAs to modulate autophagy. Specifically in breast cancer, HOTAIR has been reported to sponge miR-20a-5p, thereby upregulating HMGB1 and ATG7, key promoters of autophagy flux (Xue et al. 2016). Furthermore, HOTAIR-mediated autophagy promotes cell survival under metabolic stress, linking epigenetic regulation directly to the chemoresistance observed in high-risk patients (Li et al. 2019). Thus, HOTAIR serves as a vital scaffold orchestrating the autophagy-related network, validating its significance in our model.

Beyond phenotypic characterization, we translated these findings into clinical applications by identifying therapeutic vulnerabilities (Barretina et al. 2012). While high-risk genes like SERPINA1 were associated with broad resistance (Rajapakse et al. 2018), we intriguingly found that high MTDH expression correlates with increased sensitivity to Vincristine and Gemcitabine (Song et al. 2015; Yang et al. 2018). This finding initially appears paradoxical given MTDH’s established role as an oncogene promoting multidrug resistance. However, this discrepancy can be explained by “collateral sensitivity.” Vincristine specifically targets cells in the M-phase; thus, while MTDH drives aggressive proliferation and a high mitotic index, it inadvertently renders these rapidly dividing cells more vulnerable to anti-mitotic agents (Dumontet and Jordan 2010). This suggests that MTDH could serve as a dual biomarker: predicting poor prognosis while simultaneously indicating responsiveness to specific regimens. This discovery highlights the unique potential of our signature to guide personalized treatment, contrasting sharply with existing models that lack actionable therapeutic guidance (Deng et al. 2021; Yu et al. 2024a).

Despite these promising findings, several limitations should be acknowledged. First, this is a retrospective study based on public databases; prospective clinical trials are needed to validate the signature’s clinical utility. Second, although the validation cohort showed a clear risk trend and high AUC, larger external cohorts are required to statistically confirm the survival differences. Finally, while we propose metabolic and molecular mechanisms (such as the MTDH-Vincristine axis and metabolic competition), further experimental investigations—including in vitro and in vivo studies—are warranted to fully elucidate how these 9 genes synergistically regulate selective autophagy and immune exhaustion.

In summary, this study constructed a robust 9-gene autophagy-related signature that effectively predicts survival, delineates the immune exhaustion landscape, and identifies therapeutic vulnerabilities in breast cancer. However, as a retrospective study, inherent selection biases exist, and the molecular mechanisms underlying the MTDH-Vincristine interaction require further experimental verification. To bridge the gap between bench and bedside, future translational efforts should focus on developing a standardized 9-gene qRT-PCR diagnostic assay. This cost-effective tool could be integrated into routine pathology to complement TNM staging for refining prognosis and guiding chemotherapy selection based on MTDH expression. Although prospective multi-center validation is still warranted, our findings lay a solid foundation for developing autophagy-targeted precision medicine strategies.

## Conclusion

This study addresses the critical challenge of biological heterogeneity in breast cancer by constructing a clinically actionable 9-gene autophagy-related signature. Our findings bridge the gap between autophagic regulation and tumor immunity, revealing that high-risk tumors are characterized by a distinct “immune-exhausted” microenvironment driven by selective autophagy. Importantly, we translated these molecular features into therapeutic opportunities, identifying MTDH as a potential precision marker for targeting multidrug-resistant tumors with specific agents like Vincristine and Gemcitabine. Collectively, this signature serves not merely as a prognostic tool but as a comprehensive guide for optimizing risk assessment and personalizing therapeutic interventions in the era of precision medicine.

### Study limitations

Our study has several limitations. First, the retrospective design based on public databases (TCGA and GEO) may introduce inherent selection bias. Large-scale, multi-center prospective cohorts are required to further validate the clinical utility and robustness of our signature. Second, we acknowledge a statistical discrepancy in the external validation cohort, where the model showed robust predictive accuracy (AUC = 0.740) but marginal survival stratification significance (*P* = 0.141). This is likely attributable to the limited sample size and platform heterogeneity (RNA-seq vs. Microarray) in the GSE20685 dataset. However, the distinct separation trend in survival curves and the substantial Hazard Ratio (HR = 1.49) suggest genuine generalization potential that warrants verification in larger cohorts. Finally, our findings rely primarily on bioinformatic analyses. The lack of in vitro or in vivo experimental validation restricts our mechanistic understanding. Future studies should incorporate wet-lab experiments to verify specific molecular mechanisms and employ alternative algorithms to cross-validate the identified immune landscape features.

## Supplementary Information

Supplementary Material 1

## Data Availability

The raw data supporting the conclusions of this article will be made available by the authors, without undue reservation, to any qualified researcher.
