# Supplementary material for: Identification of a 9-gene autophagy-related signature for predicting prognosis and immune exhaustion features in breast cancer
Source: 3 Biotech. 2026 Mar 10;16(4):139. doi: 10.1007/s13205-026-04756-5 (PMC12976212; doi:10.1007/s13205-026-04756-5)
Supplement: Supplementary file 1 — Supplementary Material 1 [file 13205_2026_4756_MOESM1_ESM.docx]

| **lambda** | **Index** | **Statistic** | **Standard Error**  **(SE)** | **Number of nonzero coefficients** |  |
| --- | --- | --- | --- | --- | --- |
| lambda.min | 0.01844 | 10 | 12.789 | 0.19811 | 9 |
| lambda.1se | 0.042599 | 1 | 12.85 | 0.17222 | 0 |

**Table S1: Selection of the optimal penalty parameter (λ) for the LASSO Cox regression model.**

The optimal λ value was determined using 10-fold cross-validation with a random seed of 2022. The table presents the statistics for lambda.min (the value yielding the minimum partial likelihood deviance) and lambda.1se (the largest value within one standard error of the minimum). In this study, lambda.min (0.01844) was selected for the final model construction, resulting in the identification of 9 non-zero coefficients (signature genes).

| **variable** | **lambda.min** |
| --- | --- |
| ALDH1A3 | 0 |
| ATIC | 0 |
| AURKA | 0 |
| BAK1 | 0 |
| BIRC5 | 0 |
| CDK5 | 0 |
| CDKN2A | 0 |
| CISD2 | 0 |
| CXCR4 | 0 |
| DAB2IP | 0 |
| DCN | 0 |
| DENND3 | 0 |
| DIRAS3 | 0 |
| DYNLT1 | 0.112818201 |
| SNCAIP | 0 |
| TP63 | -0.029657883 |
| HSP90AA1 | 0.008098093 |
| PPP1R15A | 0 |
| XBP1 | 0 |
| SEC23B | 0 |
| HSPA8 | 0.043024411 |
| GAPDH | 0 |
| EPAS1 | 0 |
| VAMP8 | 0 |
| NR1D1 | 0 |
| KDR | 0 |
| GFAP | 0 |
| PRKAA1 | 0 |
| LPIN1 | 0 |
| MYC | 0 |
| GABARAPL1 | 0 |
| ERBB2 | 0 |
| PARP1 | 0 |
| SNCA | 0 |
| MTDH | 0.096871209 |
| FOXO1 | 0 |
| HSPB8 | 0 |
| NRG1 | -0.06469771 |
| KCNB1 | 0 |
| RCAN1 | 0 |
| PLEKHF1 | 0 |
| SNRPD1 | 0 |
| RAB8A | 0 |
| SNRPE | 0 |
| P4HB | 0 |
| MAPT | 0 |
| SERPINA1 | -0.08356644 |
| MAP1LC3C | 0 |
| VDAC1 | 0.122679575 |
| MEG3 | 0 |
| HOTAIR | 0.030407702 |
| NEAT1 | 0 |
| MALAT1 | 0 |

**Table S2:Nine genes selected through 10-fold cross-validation**

| **Characteristics** | **Total**  **(N)** | **HR(95% CI) Univariate analysis** | **P value Univariate analysis** | **HR(95% CI) Multivariate analysis** | **P value Multivariate analysis** |
| --- | --- | --- | --- | --- | --- |
| Pathologic T stage | 1083 |  |  |  |  |
| T1 | 277 | Reference |  | Reference |  |
| T2 | 631 | 1.334 (0.889 - 2.003) | 0.164 | 1.243 (0.791 - 1.955) | 0.346 |
| T3&T4 | 175 | 1.931 (1.208 - 3.088) | 0.006 | 1.460 (0.851 - 2.504) | 0.169 |
| Pathologic N stage | 1067 |  |  |  |  |
| N0 | 516 | Reference |  | Reference |  |
| N1 | 358 | 1.947 (1.322 - 2.865) | < 0.001 | 1.779 (1.192 - 2.653) | 0.005 |
| N2 | 116 | 2.522 (1.484 - 4.287) | < 0.001 | 2.236 (1.291 - 3.875) | 0.004 |
| N3 | 77 | 4.191 (2.318 - 7.580) | < 0.001 | 3.551 (1.888 - 6.676) | < 0.001 |
| ERBB2 | 1086 |  |  |  |  |
| Low | 542 | Reference |  |  |  |
| High | 544 | 0.986 (0.717 - 1.356) | 0.930 |  |  |
| ERBB3 | 1086 |  |  |  |  |
| Low | 542 | Reference |  |  |  |
| High | 544 | 1.066 (0.774 - 1.467) | 0.696 |  |  |
| RiskScore | 1066 | 2.718 (1.891 - 3.907) | < 0.001 | 2.718 (1.891 - 3.907) | < 0.001 |

**Table S3. Univariate and multivariate Cox regression analysis of the prognostic signature and clinical features in the TCGA cohort.**
